# Systems-based approaches to study immunometabolism

**DOI:** 10.1038/s41423-021-00783-9

**Published:** 2022-02-04

**Authors:** Vinee Purohit, Allon Wagner, Nir Yosef, Vijay K. Kuchroo

**Affiliations:** 1 Evergrande Center for Immunologic Diseases and Ann Romney Center for Neurologic Diseases, Brigham and Women’s Hospital, Harvard Medical School, Boston, MA 02115 USA; 2 Broad Institute of MIT and Harvard, Cambridge, MA 02141 USA; 3 Department of Electrical Engineering and Computer Science, University of California, Berkeley, CA 94720 USA; 4 Center for Computational Biology, University of California, Berkeley, CA 94720 USA

**Keywords:** Immunometabolism, Metabolic techniques, GSMM, Metabolic modeling, Systems biology, Immunology, Cell biology

## Abstract

Technical advances at the interface of biology and computation, such as single-cell RNA-sequencing (scRNA-seq), reveal new layers of complexity in cellular systems. An emerging area of investigation using the systems biology approach is the study of the metabolism of immune cells. The diverse spectra of immune cell phenotypes, sparsity of immune cell numbers in vivo, limitations in the number of metabolites identified, dynamic nature of cellular metabolism and metabolic fluxes, tissue specificity, and high dependence on the local milieu make investigations in immunometabolism challenging, especially at the single-cell level. In this review, we define the systemic nature of immunometabolism, summarize cell- and system-based approaches, and introduce mathematical modeling approaches for systems interrogation of metabolic changes in immune cells. We close the review by discussing the applications and shortcomings of metabolic modeling techniques. With systems-oriented studies of metabolism expected to become a mainstay of immunological research, an understanding of current approaches toward systems immunometabolism will help investigators make the best use of current resources and push the boundaries of the discipline.

## Introduction

*IIl y a des systèmes vivants; il nʼy a pas de matière vivante*.

*There are living systems; there is no living matter*.

These words of Nobel laureate Dr. Jacques Monod [1] are increasingly relevant with our improved appreciation of the complexity of biology at the cellular and organismal levels. Today, systems biology is a well-established branch of scientific interrogation, but in the last century, unlike its contemporary sciences such as physics, biology was focused on reductionist approaches and deciphering cause and effects attributable to individual molecules, cells, and parts of the genome. However, as Monod expressed, reductionist studies are insufficient in light of the interrelatedness of biological components as systems.

With the advent of high-dimensional sequencing techniques, heterogeneity within immune cell populations has become more evident [2–8]. Gury-BenAri et al. performed the single-cell analysis of innate lymphocytes (ILCs) from the mouse intestine to determine seven “ILC states” (ILC1a, ILC1c, ILC1d, ILC2a, ILC2c, ILC2d, and ILC3a) to be highly responsive to microbial colonization [4]. Similarly, another study identified three functionally distinct subsets of ILC3s: NKp44^+^ ILC3s, CD62L^+^ ILC3s, and HLA-DR^+^ ILC3s [2]. Single-cell analysis has also revealed the transcriptional and functional spectrum within T cell subtypes such as T helper 17 (Th17) [3] and T regulatory (Treg) cells [9]. The balance between pathogenic and nonpathogenic Th17 cells (Th17p and Th17n cells respectively) shapes tissue inflammation [10–12]. Gaublomme et al. used scRNA-seq to identify multiple genes that regulate this balance, including CD5-like (*Cd5l*), that were not found at the population RNA-seq level. Genetic ablation of these genes had a strong biological impact on the development of disease in murine model of experimental autoimmune encephalomyelitis (EAE) [3]. These examples illustrate the potential of systems methods to uncover the heterogeneous spectrum of immune cells and to aid the discovery of novel targets for potential therapeutic intervention that might not otherwise be evident at the population level [13].

Similar to immunity, cellular metabolism is also systematically regulated. In fact, the two branches of biology have been connected since Otto Warburg discovered that activated leukocytes have increased aerobic glycolysis and not oxidative metabolism [14]. Metabolism fulfills three basic needs of an organism, i.e., the generation of energy in the form of ATP, the maintenance of redox potential by the generation of NAD(P)H, and the synthesis of macromolecules [15, 16]. All living systems, including immune cells, take in nutrients and utilize them to fulfill these needs. While it is common to abstract metabolism as a set of pathways and cycles, cellular metabolism comprises a complex network of reactions that are highly interconnected through common substrates, products, cofactors, and catalyzing enzymes. A limited perturbation target may therefore alter the entire network to maintain metabolic homeostasis. To study immunometabolism, one has to consider the dynamic nature of immune cells, heterogeneity within immune cell populations, and rapid changes in disease environments. Here, we review system-based approaches to interrogate immunometabolism and introduce mathematical modeling tools that promise to advance the field.

## Experimental approaches to interrogate immunometabolism

The foundation of our current understanding of immune cell metabolism is based on cell-focused approaches. Classically, the uptake of 2-deoxy-2-[(7-nitro-2,1,3-benzoxadiazol-4-yl) amino]-d-glucose (NBDG) or radiolabeled deoxyglucose has been used as an indicator of glycolysis, a method also implemented in clinics for diagnosis of cancer in the form of 18-fluorodeoxyglucose positron emission tomography (FDG-PET). Reinfeld and Madden et al. recently applied FDG-PET to identify myeloid cells, and not tumor cells, as the principal consumers of glucose in a tumor microenvironment, contrary to the established paradigm [17]. Extracellular flux analysis (EFA) can also be performed for the quantification of cellular respiration and indirect assessment of glycolysis through the measurement of lactate release. Corroborating Warburg’s seminal findings in lymphocytes in the 1950s, recent work has shown that T cell receptor activation dramatically increases both the extracellular acidification rate (ECAR) and the oxygen consumption rate (OCR) [18], which are indicative of increased glycolysis and aerobic mitochondrial respiration, respectively. Similarly, Tregs and Th17n cells have an increased OCR and reduced ECAR compared to Th17p cells [19].

Flow cytometry, which is used to detect soluble and cell surface proteins, can also be used to measure metabolic parameters in immune cells, such as glucose uptake [20, 21], lipid levels by BODIPY [22], and FITC-based cysteine uptake assays [23]. This widely implemented technique is also foundational to a multiplexed and systems tool for the interrogation of immune cell metabolism using cytometry by time of flight (CyTOF) mass spectrometry. CyTOF quantifies up to 40 antibody-based metabolic proteins, cell surface markers, and cytokines to allow parallel characterization of the immune and metabolic states of an individual cell. This can be applied not only to understand the metabolic [24–26] and effector states [27, 28] of immune cells but also to determine epigenetic changes such as acetylation [29]. Hartmann et al. recently developed a mass-cytometry-based method called single-cell metabolic regulome profiling (scMEP) that determines cell phenotype by identifying metabolic regulators [26]. Combining scMEP with multiplexed ion beam imaging by the time of flight (MIBI-TOF), the authors determined that CD39^+^ PD1^+^ T cells are spatially restricted to the tumor-immune boundary, implicating a broader, noncheckpoint-dependent mechanism of immune exclusion in human colorectal cancers. Similarly, Levine et al. implemented mass cytometry to determine metabolic regulators of CD8^+^ T cell activation after *Listeria monocytogenes* infection in vivo [25]. The authors observed that early activated CD8^+^ T cells have an increase in both, glycolysis proteins such as glyceraldehyde 3-phosphate dehydrogenase (Gapdh) and glucose transporter 1 (Glut1/gene name *Slc2a1*), and mitochondrial protein such as ATP synthase F1 subunit alpha (Atp5a), which was confirmed functionally by EFA and MitoTracker staining [25]. Another flow cytometry-based strategy is Met-Flow, which determines the metabolic state of a cell based on the expression levels of rate-limiting enzymes of principal metabolic pathways. For example, high levels of Glut1 and hexokinase 1 are indicative of increased glycolysis, and glucose-6-phosphate dehydrogenase (G6PD) of increased oxidative pentose phosphate pathway [30]. Of note, protein synthesis is also a major consumer of energy such that changes in protein synthesis, especially after metabolic interventions, are an informative readout. Applying this understanding, Argüello et al. developed SCENITH (single-cell energetic metabolism by profiling translation inhibition), which quantifies protein translation with puromycin staining as a surrogate to assess metabolic changes in heterogeneous cell populations at single-cell resolution. Using this technique, Lopes et al. unveiled the metabolic dichotomy between γδ T cell subsets in EO711 and MC38 tumor models and determined that whereas IFN^+^γδ T cells rely on glycolysis, IL17^+^γδ T cells depend on mitochondrial metabolism [31]. The flow cytometry readout can be made more comprehensive by the inclusion of markers for DNA and RNA along with proteins [32]. All these experimental techniques are validated tools to study metabolism and provide substantial insights into the metabolic dependencies of immune cells. However, the assessment of immunological proteins in cells grown under nonphysiological conditions and the fact that multiplexing by CyTOF is limited to only 40 markers restrict the applicability of these methods for the detection of the metabolic heterogeneity of immune cells.

Unlike flow-based approaches, RNA sequencing is comprehensive and has no intrinsic limit on the number of marker readouts per cell. Indeed, scRNA-seq data have been instrumental in understanding heterogeneity and identifying novel metabolic regulators of immune cells. Through insights provided by scRNA- seq, we identified *Cd5l*, a regulator of lipid metabolism, as a critical mediator of Th17 cell pathogenicity [3, 33]. We discovered that high Cd5l in Th17n cells shifts cellular lipid profile by increasing polyunsaturated fatty acids (PUFAs) while reducing saturated fatty acids (SFAs) and cholesterol biosynthesis. Genetic ablation of *Cd5l* promotes pathogenicity in Th17 cells by increased cholesterol metabolites and the activation of RORγT [33], the master transcription factor of Th17 cell differentiation. Similarly, based on single-cell analysis, Rivadeneira et al. found that vaccinia-virus-infected melanoma tumors have an increased influx of new TIM3^high^ PD1^mid/low^ CD8^+^ T cells that, despite appearing non-exhausted, are metabolically insufficient, as indicated by reduced mitochondrial content and increased glycolysis [34]. Local administration of leptin, an adipokine with metabolic effects [35], increased basal oxygen consumption and mitochondrial reserve, thereby increasing the inflammatory capacity and antitumor effector functions of T cells. In a separate study, Ringel et al. performed the single-cell analysis of tumor-infiltrating CD45^+^ lymphocytes from mice fed control and high-fat diet [36]. The authors reported that a high-fat diet reprograms tumor metabolism, specifically fat utilization between tumor and CD8^+^ T cells. Single-cell analysis of human melanoma, and head and neck cancers, revealed the metabolic programs governing the tumors and non-tumor populations [37]. Similarly, leveraging scRNAseq analysis Fernández-García et al. identified asparagine synthetase (*Asns*) as a dynamic regulator of CD8^+^ T cell effector function during viral infections, downregulation of which polarizes these cells to a memory phenotype [38]. These examples highlight the efficacy of scRNA-seq as a tool to identify new metabolic targets that regulate the T cell response in autoimmunity and cancer.

The quantification of intracellular and extracellular metabolites by nuclear magnetic resonance-based (NMR) and mass spectrometry (MS)-based metabolomics can serve as orthogonal confirmation of the results of metabolic gene expression analyses based on sequencing data. NMR lacks the resolution of MS-based methods in identifying a specific metabolite but is nondestructive to samples and is quantitative. By doing NMR analysis of T lymphocytes obtained from septic shock patients Venet et al. found that these T cells had impaired glycolysis and ATP production, which was corrected upon treatment with IL7 [39]. Compared to NMR, MS-based metabolomics is more widely implemented in the study of cellular metabolism due to its greater sensitivity and resolution. It is usually done in tandem with chromatography-based separation of metabolites and hence called liquid chromatography-mass spectrometry (LC-MS) or gas chromatography-MS (GC-MS). We and others have applied LC-MS-based metabolomics to study the metabolic basis of Th17 cell differentiation and fate [33, 40, 41]. We identified that Th17p cells depend on polyamine biosynthesis, which when inhibited, shifts Th17 cells into a nonpathogenic/regulatory state and ameliorates EAE [19].

The steady-state metabolome is a snapshot of metabolite levels at a given time and thus provides an incomplete picture. Appreciating the dynamics of metabolism warrants the assessment of material flow per unit time, i.e., metabolic flux [41]. Ron-Harel et al. labeled naive and activated T cells with ^13^C_3_-d-serine to measure the incorporation in de novo purine biosynthesis metabolites [42]. The authors identified that the knockdown of the mitochondrial serine metabolizing enzyme *Shmt2* resulted in an accumulation of the metabolites α-phosphoribosyl pyrophosphate (α–PRPP), glycineamide ribonucleotide (GAR), 5-aminoimidazole-4-carboxamide ribonucleotide (AICAR), and succinylaminoimidazole carboxamide ribose-5′-phosphate (SAICAR) upstream of 10-formyl THF incorporation. Based on dynamic labeling, the authors identified the mitochondrial folate pathway as critical for supplying one-carbon units for de novo purine synthesis in activated T cells [42]. Similarly, using U-^13^C_6_-glucose (glucose labeled on all six carbons with ^13^C) tracing followed by GC-MS-based analysis, Wu et al. assessed the ^13^C incorporation kinetics of pyruvate, lactate, serine, glycine, alanine, and citrate in olfactory receptor *Olfr2-*knockout, glucose-6-phosphate isomerase 1 (*Gpi1)-*deficient, and koningic acid (KA)-treated Th17p and Th17n cells [43]. Stable isotope labeling (SIL) can also be performed in vivo through the slow infusion of nutrients such as U-^13^C_6_-glucose to determine the fate of downstream metabolites [44]. ^13^C-based SIL to assess murine T cell metabolism during an active immune response has shown that effector CD8^+^ T cells adopt a distinct metabolic profile in vivo, and produce little lactate and oxidize most of their glucose through the TCA cycle [45]. Further, pyruvate dehydrogenase mediates entry of pyruvate to the TCA cycle in vivo unlike in vitro environment where it is mediated by pyruvate carboxylase. Finally, the fate of glucose is different in T cells during different phases and is associated with an increased dependence on serine biosynthesis during the effector phase [45].

Mass spectrometry can be employed to study both metabolome and proteome. Applying this strategy, Geiger and colleagues discovered that l-arginine concentrations regulate T cell proliferation, differentiation, and survival. These authors found that an increase in l-arginine concentrations promotes oxidative phosphorylation (OXPHOS) and central memory T cell formation with enhanced antitumor efficacy [46]. Similarly, LC-MS-based quantitative analysis of the proteomes of murine naive CD4^+^ and CD8^+^ T cells, revealed that sensing of environmental cues and metabolic reprogramming of T cells depend on activation by cognate antigens [47]. Further, mTORC1 inhibition has a cell context-specific effect on cell cycle progression such that it is prominent in antigen-activated naive CD4^+^ and CD8^+^ cells but not in effector cells [47]. In another study, by applying tandem mass tag method and two-dimensional liquid chromatography-tandem mass spectrometry (LC/LC-MS/MS) followed by integrated in silico analysis, Tan et al. determine the signaling and bioenergetic mediators of T cell exit from quiescence [48]. The authors found that the absence of cytochrome oxidase 10 (Cox10), an accessory factor for the assembly of mitochondrial complex IV, reduced Ifn-γ^+^ TNF-α-producing Th1 cells in mice infected with ovalbumin-expressing recombinant *Listeria monocytogenes* (LM-OVA) [48]. Using a different approach to compare naive and activated T cells, Wolf and colleagues observed that even though naive T cells do not rely on glycolysis these cells have a large reservoir of glycolytic enzyme (amounting to 11% of the total cytosolic proteins) as a mechanism of T cell preparedness for activation [49]. By pulsed SILAC (stable isotope labeling of amino acids in cell culture)-based approach, these authors found that upon activation T cells increase the activity as well as turnover of major glycolytic proteins such as lactate dehydrogenase (Ldha), Gapdh, aldolase A (Aldoa), and phosphoglycerate kinase 1 (Pgk1) that feed the altered metabolic demands of activated cells [49]. Mass spectrometry can also be applied to measure the in vitro activity of enzymes. Ghergurovich et al. performed LC-MS-based monitoring of 6-phosphogluconate production by recombinant human G6PD in vitro to assess the activity of the pentose phosphate enzyme G6PD in T cells and macrophages [50].

The CRISPR revolution has provided another means to assess the effect of targeting specific genes or a library of genes on immune cell metabolism and phenotype. An elegant example of this is the recent work by Huang and colleagues that leverages CRISPR-Cas9-based in vivo pooled screening to determine metabolic regulators of Klrg1^−^CD127^+^ or Klrg1^lo^CD127^hi^ memory precursor (MP) and Klrg1^+^CD127^−^ or Klrg1^hi^CD127^lo^ terminal effector (TE) CD8^+^ T cells under the acute lymphocytic choriomeningitis Armstrong strain (LCMV) infection model [51]. The authors observed that the loss of amino acid transporters *Slc7a1* and *Slc38a2* promoted MP CD8^+^ T cell formation and persistence by regulating amino acid levels and mTORC1 activity. The same screening also uncovered the GDP-fucose protein O-fucosyltransferase 1(Pofut1), as the key regulator of a different subset of Klrg1^hi^ Cxcr3^lo^ CD127^lo^ terminal effector (TE’) T cell population. *Pofut1* deletion reduced the TE’ frequency but improved the accumulation of Cxcr3^hi^CD127^lo^ effector T cells in a poised state (T_INT_) that have cytotoxic features. Application of CRISPR-Cas9 screening followed by in vivo validation revealed de novo synthesis of phosphatidylethanolamine as a metabolic and posttranscriptional regulator of CXCR5 protein stability and membrane localization in T follicular helper cells [52]. In another study, using CRISPR-Cas9-based screening of metabolism-associated factors, Wei et al. identified zinc finger CCCH-type containing 12 A or *Zc3h12a* (also known as Regnase-1) as a major negative regulator of antitumor response [53]. By performing secondary in vivo genome-scale CRISPR screening, these authors identified Basic Leucine Zipper ATF-Like Transcription Factor (Batf)-mediated mitochondrial oxidative metabolism as a regulator of increased effector response in Regnase-1 null CD8^+^ T cells. Simultaneous inhibition of both Regnase-1 and Batf diminished the increased mitochondrial mass, membrane potential, and effector function of Regnase-1 null CD8^+^ T cells [53]. All these studies illustrate how CRISPR-based screening is a powerful tool to determine functional and phenotypic regulators of immune cell metabolism.

Integrated approaches that combine high throughput sequencing with traditional biochemical, metabolic, and molecular biology techniques have also been developed to interrogate the metabolism of immune cells. An example is Ins-seq, which integrates massively parallel measurements of scRNA-seq and intracellular protein activity [54]. Utilizing this method on bone marrow cultures stimulated with LPS, Katzenelenbogen et al. identified Trem2 as a marker of immunosuppressive myeloid cells in the tumor microenvironment [54]. Another technique combining scRNA-seq data with the spatial distribution of immune cell population is Zipseq, which has been used in tandem with SCENITH to determine both the location and the metabolic traits of immune cells, as used for the examination of CD8^+^ T cell populations in the tumor microenvironment [55].

In summary cell-based, cytometry-based, and high-dimensional techniques are the workhorses of immunometabolic interrogations, the latter being the foundation of systems methods currently being implemented. Giving examples of each, we have attempted to provide a brief introduction to these approaches. For the details of these methods, the readers are referred to recent excellent reviews in the field [28, 56–60].

## Computational metabolic modeling complements experimental approaches

It is challenging to holistically characterize cellular metabolic states with direct metabolic assays. Assays such as glucose uptake or EFA or flow-cytometry-based methods depend on isolated cells that are cultured or sorted under nonphysiological conditions before or during analysis, effectively causing a loss or damage of metabolites. Metabolomics assays, although high throughput, require cell numbers that make it challenging to study small immune populations, especially in vivo. In addition, metabolomic assays are unable to measure in parallel and distinguish a large enough number of molecules to be considered comprehensive. These gaps can be effectively addressed by functional genomic methods, which may not provide direct metabolic readouts, but their advantages effectively complement direct metabolic assays. When used in tandem, gene/metabolite enrichment analyses can identify genes/metabolites significantly altered in experimental datasets by leveraging the power of known gene ontologies and established metabolic databases such as KEGG [61], thereby providing insights into altered metabolism, function, and heterogeneity among immune cells. However, the metabolic network is highly interconnected, and fragmentation into pathways inevitably loses some of the ways in which pathways can interact and affect one another. Moreover, pathway composition may not be the same between different tissues or in disease states. Therefore, enrichment analysis of predefined pathways cannot account for the full complexity of the metabolic network.

**Fig. 1:**
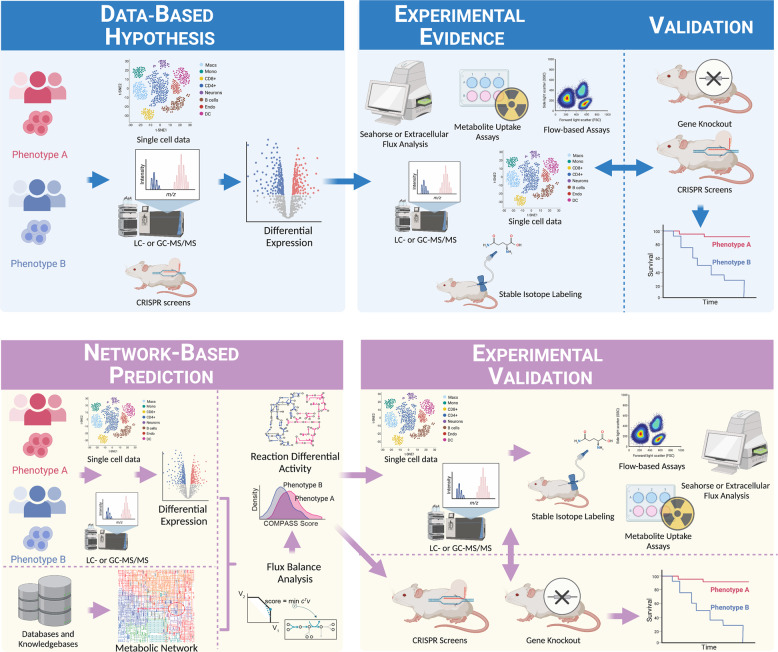
Network-based methods complement and add to enrichment-based workflows for interrogating immunometabolism. Top panel: High-throughput data, such as transcriptomics, metabolomics, or systemic CRISPR screens, are used to generate data-driven hypotheses in the form of differentially expressed targets [119, 120]. Pathways and gene sets are knowledge-based representations of shared biological activity derived from established gene ontologies and databases (e.g., GO [121, 122] and KEGG [61]). Subsequent experiments validate and refine the data-driven hypotheses, add mechanistic insight, and may lead to another cycle of high-throughput data collection and analysis. Bottom panel: Network-based approaches augment enrichment-based approaches. Biological networks, such as genome-wide metabolic networks, are generated from the annotated genome of a species of interest together with functional genomic data and computational gap-filling where appropriate [104–106]. Network algorithms, such as flux balance analysis for genome-scale metabolic models, integrate global information agnostic of pathway divisions and can therefore predict targets that will not be prioritized based on the workflow described above. Similar to pathway- and gene-set-based approaches, networks organize extant knowledge and allow the contextualization of gathered high-throughput data within extant knowledge bases. However, network approaches may capture systemic effects that are missed by pathway-focused approaches.

A different way to approach this is by utilizing metabolic models that translate available knowledge of the topology and stoichiometry of the metabolic network of a species into mathematical models. These models allow a structural analysis of the metabolic network [62, 63] and, importantly, in silico predictions of metabolic fluxes, which can then be confirmed by biochemical and metabolic techniques. They offer a powerful way to contextualize high- dimensional omics data, such as scRNA-seq data, within species- specific metabolic knowledge. Metabolic modeling can be steady- state or kinetic and leverages the strength of computation to predict cellular metabolism. Of note, metabolic models are simplified maps for explaining and predicting biological phenomena. Hence, although metabolic approaches augment standard enrichment-based approaches, the resultant predictions also need to be confirmed and revised by traditional experimentation techniques. This complementarity of two approaches warrants the need for current methods to work in tandem with metabolic modeling to develop a holistic understanding of cellular metabolism in the immune system. Figure 1 provides a summary of the workflow of prevalent non-network-based approaches and metabolic modeling-based methods. In the following sections, we will introduce the basics of current metabolic modeling approaches to study cellular metabolism, provide a basic workflow of genome-scale metabolic models (GSMMs), and summarize the application and shortcomings of metabolic models to interrogate immunometabolism.

## Genome-scale metabolic models as tools to study immunometabolism

Metabolic modeling of cellular metabolism broadly falls into one of two branches: kinetic vs. steady-state approaches [64]. Kinetic methods directly model metabolite concentrations as a function of time and reactions that produce or consume them as ordinary differential equations. Challenges to this approach include incomplete knowledge of reaction kinetics, especially in vivo, and computational intractability at scale. Hence, kinetic approaches have thus far been applied to unicellular organisms such as *E. coli* [65] and yeast [66, 67] and to simpler systems such as human red blood cells, which lack mitochondria-dependent metabolic pathways [68, 69]. In contrast, steady-state approaches model genome-scale metabolic networks by simplifying the assumption of a biochemical steady-state and consequently do not directly model metabolite concentrations [70]. Their network-wide metabolic coverage, which allows accounting for subcellular metabolite compartmentalization and cell-to-cell metabolic exchanges, has been leveraged to study human metabolism in diverse contexts and to perform in silico combinatorial perturbation experiments [71, 72]. In this review, we focus on one of the main computational frameworks for steady-state metabolic modeling, GSMM, which offers two advantages in the study of immunometabolism. First, GSMMs can be contextualized with single-cell genomics, which is advantageous in the study of rare and highly heterogeneous cells, as in the case of the immune system. Second, GSMMs allow in silico hypothesis generation, for example, via a network-wide in silico search of metabolic targets modulating an immune cell phenotype.

**Fig. 2:**
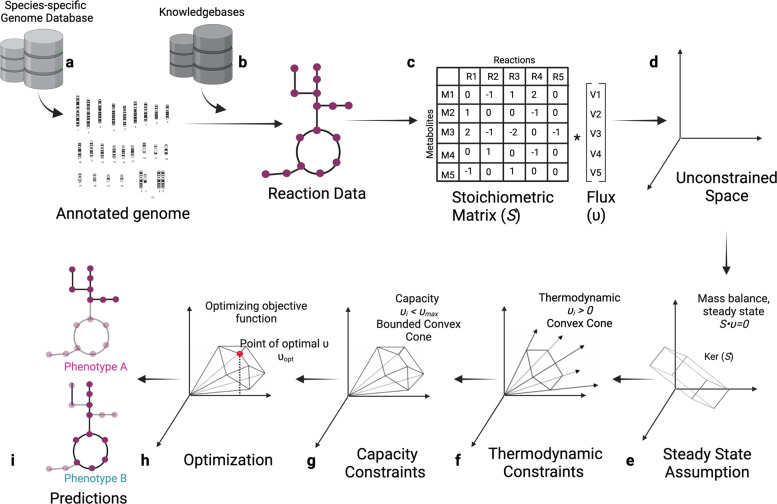
Schematic overview of a constraint-based modeling approach to study metabolism. **a** Annotated genes from a species of interest are combined with metabolic knowledge bases to generate a draft for the metabolic reactions available to a cell. **b** This draft is refined based on existing knowledge bases, and computational gap filling [104–106] is applied to ensure desired properties, such as the ability to generate ATP from a given substrate. **c** The product of this phase is a stoichiometric matrix (S) wherein entries are the stoichiometric coefficient of a particular metabolite (row) in a particular reaction (column). Reactions that have only negative or positive entries are exchange reactions that allow metabolite intake into and secretion out of the system (e.g., R5 in the illustrated matrix). **d**, **e** Imposing the assumptions of mass balance and of biochemical steady-state (i.e., constant metabolite concentrations) leads to a feasible space of metabolic flux distributions *v* (mathematically, this is the kernel of the stoichiometric matrix, namely, the solution space of *S* · ν = 0). The addition of (**f**) thermodynamic and (**g**) capacity constraints further restrict the feasible flux distribution space into a convex cone and bounded convex cone, respectively [71, 123, 124]. **h** The optimization of objective functions is used to detect mechanistically relevant flux distributions (i.e., the assignment of predicted flux for each reaction). The optimization of the pertinent objective (e.g., synthesis of biomass molecules) allows finding the vertices of the convex cone (namely, specific flux distributions) of interest, although the system is often underconstrained, and as a result, the solution to the optimization problem is nonunique. **i** The final outcome is predicted flux distributions, namely, the assignment of flux values to each reaction, that achieve the optimum of the stated objective, subject to the stated constraints. Often, the optimization function and/or constraints are informed by empirical high-throughput data, such as gene expression of different phenotypes/cells as denoted in the figure.

Foundational to any GSMM is the generation of a well-curated organism-specific metabolic network based on omics data such as, proteomics, transcriptomic, and metabolomics; published literature; and reference network reconstructions such as, BiGG (Biochemical Genetic and genomic knowledgebase) [73], VMH (Virtual Metabolic Human) (https://www.vmh.life/), ModelSeed (https://modelseed.org/), and MetaNetX (https://www.metanetx.org/). Notably, GSMMs encode more than the association between reactions with substrate and product metabolites. They contain information on associating metabolic reactions with genes that code the enzymes catalyzing these reactions [74] and reaction stoichiometry in matrix format (*S*) [75] (Fig. 2). Stoichiometry, together with the assumptions of mass balance and steady-state, defines a space of feasible flux distributions (v), i.e., the assignment of flux value through to every reaction in the network. This solution space can be constrained further, for example, by the imposition of nutrient availability constraints or reaction irreversibility due to thermodynamic considerations (Fig. 2), upon which further condition- or outcome-specific constraints can be applied. In the next step, particular flux distributions may be selected based on constrained optimization of a desired cellular property, such as biomass production or the synthesis of a desired metabolite [76, 77]. These steps are carried by Flux Balance Analysis (FBA) algorithms, which use constrained optimization, such as linear or mixed-integer linear programming to be applied to predict the direction and flux of metabolic reactions that support a given biological objective, such as cell growth, macromolecule biosynthesis, and the regulation of redox balance. The steps of creating a GSMM and constraint-based analysis are shown in Fig. 2, and the basics of the latter are enumerated in Box 1. GSMMs have been used to study mammalian cells, including red blood cells (RBCs) [68], cancer cells [78, 79], hepatocytes [80, 81], adipocytes [82, 83], and neurons [84, 85]. GSMMs have also been applied to interrogate immune cell metabolism. One early example was the creation of a GSMM for the RAW 264.7 macrophage cell line [86], which was used to identify metabolic regulators of immune activation and immune suppression in macrophages. Based on this, the authors identified glutamine/glutamate utilization in de novo nucleotide synthesis as a key pathway downregulated during LPS-mediated activation of macrophages [86]. Hörhold et al. used FBA modeling and gene set enrichment to study macrophage regulation and identified nine top-ranking transcription factors that regulate an M1 to M2 phenotype switch, which were then confirmed by in vivo studies [87].

To predict the cellular state with greater accuracy, high-throughput data such as transcriptomics, metabolomics, or proteomics can be used to limit the solution space of constraint-based metabolic models. Among the available high-dimensional data, scRNA-seq is unrivaled in comprehensiveness by covering all enzymes in the entire genome of an organism. Furthermore, scRNA-seq is cost-efficient and can be compared to a high volume of available datasets. With an increased impetus to develop human-cell-based datasets such as the Human Cell Atlas [88], the potential of RNA expression in metabolic prediction is increasing. The novel avenues afforded by scRNA-seq (unlike bulk sequencing) require new methods to account for its characteristics and to exploit the novel opportunities it allows. This led us to develop Compass [19], an FBA algorithm that models the metabolic flux states of individual cells based on single-cell data. Intuitively, Compass determines the alignment of the cell’s global metabolic program, reflected in its transcriptome, without carrying a high flux on a particular reaction. This is quantified by the Compass score for the reaction, which we interpret as a proxy for the activity of the reaction in cells [19]. Compass-predicted metabolic profiles can then be used to identify differentially active metabolic reactions between cell types or reactions associated with continuous spectra of cellular states within a dataset. Notably, Compass takes a global view of the metabolic network and does not partition it to predetermined metabolic pathways, allowing it to predict novel connections between a phenotype and ancillary metabolic pathways or between seemingly distant metabolic reactions. In our study, we demonstrated the utility of Compass in the assessment of the metabolic state of individual cells in a disease to identify new metabolic targets to ameliorate the disease [19]. This algorithm can be applied either to single cells or to groups of cells (metacells [89, 90] or pseudobulks) and produces a quantitative metabolic profile for each cell or subgroup that can be calculated based on their transcriptomic profile. A summary of Compass is provided in Box 2.

Box 1 Basics of flux balance analysis *Stoichiometric matrix (S)*

The stoichiometric coefficients are the multiplicative factors added to chemical formulas to preserve mass balance. A stoichiometric matrix *S* describes the set of possible metabolic reactions in the system. Its rows correspond to metabolites, its columns correspond to reactions available to the cell, and entries hold the stoichiometric coefficients of a metabolite in a reaction.

*Metabolic flux (v)*

A metabolic flux is an instantaneous rate at which a chemical reaction occurs and is measured in units of particles per time and volume. Let *x* be vectors of metabolite concentrations in the system (as a function of time *t*) and *v* be the vector of metabolic fluxes. Then:

$$S \cdot v = \frac{{dx}}{{dt}}$$

FBA algorithms [125] assume a metabolic steady-state, i.e.,

$$S \cdot v = 0$$

Equivalently, we limit the space of feasible flux distributions *v* to ker (S) (Fig. 2e).

In addition to the steady-state assumption, the vector of flux values per reaction is subject to thermodynamic (e.g., reaction irreversibility) and chemical (e.g., nutrient availability) constraints, which further limit the space of feasible flux distributions (Fig. 2f, g) [125]. FBA then employs constraint-based optimization to detect points of interest in the high-dimensional flux distribution space (Fig. 2h, i) [76, 109,126–128].

Box 2 Compass: an FBA tool to study immunometabolism Compass is an FBA algorithm that uses single-cell transcriptomic profiles to produce a quantitative profile for the metabolic state of single cells. Even though the mRNA expression of enzymes is not an accurate proxy for their metabolic activity, a global analysis of the metabolic network (as enabled by RNA-seq) in the context of a large sample set (as offered by single-cell genomics) coupled with strict criteria for hypothesis testing provides an effective framework for predicting cellular metabolic states.

The first step of Compass is agnostic to gene expression and computes, for every metabolic reaction *r*, the maximal flux $v_r^{opt}$ it can carry while imposing only stoichiometry and mass balance constraints. Next, Compass assigns every reaction in every cell a penalty inversely proportional to the mRNA expression associated with the enzyme(s) catalyzing the reaction in that cell. Finally, for every reaction *r* and every cell, Compass finds a flux distribution (an assignment of flux values to every reaction in the network) that minimizes the overall penalty incurred while maintaining a flux of at least $\omega \cdot v_r^{opt}$(we used *ω* = 0.95) through *r*. The additive inverse of this penalty term is the reaction score.

The use of genome-scale metabolic networks allows the entire metabolic transcriptome to impact the computed score for any particular reaction, rather than just the mRNA coding for the enzymes that catalyze it. This reduces the effect of instances where mRNA expression does not correlate with metabolic activity and of scRNA-Seq dropouts [129]. Compass further mitigates data sparsity effects through information sharing on a *k*-nearest neighbors graph, similar to other scRNA-Seq algorithms [89,130–133]. Single-cell gene expression sparsity can also be effectively addressed by pooling cells together based on data-driven heuristics (e.g., metacells [89] or micropools [134]) or based on the experimental design (e.g., pooling together experimental replicates to create pseudobulks).

The resulting quantitative profile of the metabolic state of a cell, represented by a reaction score matrix, is subjected to further analyses, which can be (a) hierarchical clustering of metabolic reactions as metareactions, (b) computing differential expression of reactions, (c) the correlation of reactions with phenotype under interrogation, and (d) the analysis of data-driven pathways [19].

## Applications of metabolic modeling in immunometabolism

**Fig. 3:**
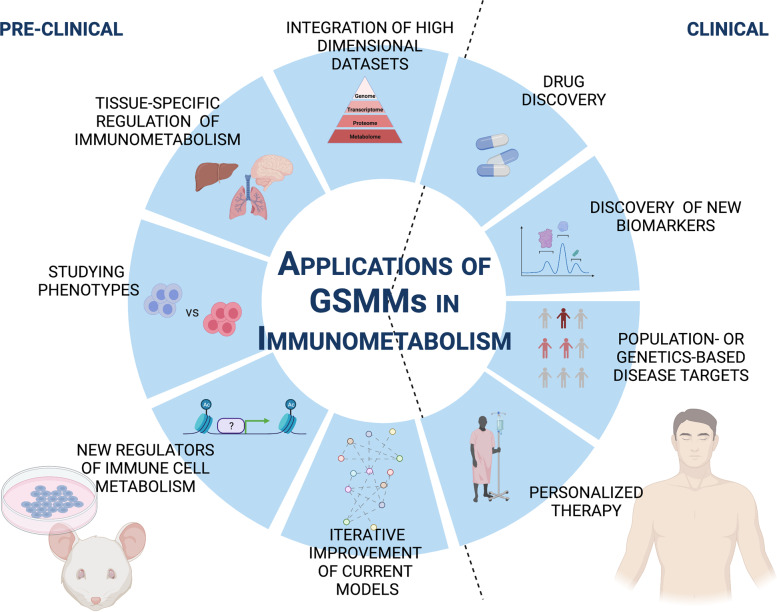
Preclinical and clinical applications of genome-scale metabolic models (GSMMs): Cartoon depiction of the current use and potential applications of GSMMs. The left panel represents the preclinical applications of GSMMs in mice and cell lines. The right panel summarizes the clinical applications in humans.

Genome-scale models can be useful tools to gain a better understanding of the metabolic requirements of rare immune cells, especially in vivo, and consequently are an important tool to study immune cells both under homeostasis and in disease. With the increasing availability of high-throughput data, metabolic models can have several potential applications in both preclinical and clinical settings (Fig. 3), a few of which are summarized here.

### Studying environment-specific metabolic dependencies of immune cells

Immune cells moving from blood or lymphoid tissue to the tissue of residence face a shift in microenvironment-derived metabolic input as well as oxygen concentrations. Angelin and colleagues demonstrated that Tregs maintain OXPHOS only under nutrient- and oxygen-replete conditions [91]. Under glucose-limiting conditions and hypoxia, as seen in cancers and inflammatory diseases, T cells rely on lactate as a preferred carbon source and direct it to mitochondrial metabolism. Not only Tregs but also effector CD4^+^ T cells within an inflammatory microenvironment, such as an arthritic joint, express lactate transporters to support their survival [92]. Simulating these conditions under artificial in vitro conditions can be difficult. Systems approaches offer a solution to this through model extraction algorithms. A context-specific genome-scale metabolic model (CSM) takes into consideration context-specific reactions or enzymes expressed in a given tissue or cell line [86, 93]. In this case, model extraction methods are applied to remove reactions deemed inactive based on observed (expression, protein, and metabolic) data, predefined metabolic functions of a tissue and knowledge from published literature. In the absence of widely available protein and enzyme activity data, various groups have successfully utilized gene expression data to generate CSMs. Zhang et al. recently applied constraint-based CSMs for scRNA-seq data to simulate NAD^+^ biosynthesis activity in seven mouse tissues [94]. Similarly, using *Caenorhabditis elegans* as a model, Yilmaz et al. implemented their new computational pipeline MERGE to identify tissue-specific metabolism in the nematode. Applying tissue-specific constraints on scRNA-seq data from various tissues of *C. elegans*, the authors found that the metabolic properties of a nematode tissue depend on the established functions of the tissue and that metabolic similarities with analogous human tissues could be identified [95]. These studies provide an excellent basis for the application of CSMs to study the tissue-specific metabolic heterogeneity of mammalian immune cells.

### Identification of novel regulators of disease

Distinct T cell subsets are associated with autoimmunity and inflammation. In EAE, an animal model of multiple sclerosis, the balance between Th17p and Th17n/Treg cells regulates the outcome and severity of disease. Using the FBA algorithm Compass, we studied the metabolic underpinnings of T cell pathogenicity in simplified in vitro settings [19]. Leveraging single-cell data, Compass predicted increased dependence of regulatory and nonpathogenic T cells on fatty acid oxidation. Additional principal components uncovered nitrogen metabolism, specifically urea cycle targets that had the capacity to modulate Th17 pathogenicity. Compass identified polyamine metabolism as a key regulator of the T cell phenotype and predicted its requirement in the generation of Th17p cells. By performing complementary in vitro and in vivo studies, we confirmed that the inhibition of polyamine biosynthesis shifts T cells to a nonpathogenic/regulatory phenotype in murine models of MS [19]. It is important to note that the outcome of GSMMs can vary based on the application of different constraints that are provided by different disease microenvironments. Therefore, different metabolic pathways could underlie the development of autoimmune diseases in different tissues.

### Integration of multiomics to increase the depth of metabolic networks

The basis of genome-scale networks is the integration of omics data to generate draft reconstructions upon which GSMMs are built. Integration approaches that can incorporate multiple omics data can enhance the depth of network-based approaches. Jha et al. developed such a network-based approach concordant with metabolomics integrated with transcription (“CoMBI-T”) that allows the integration of transcriptional and ^13^C metabolomics data. Applying this pipeline, the authors identified UDP-GlcNac and glutamine metabolism as characteristic features of M2 macrophage polarization. Furthermore, an inflammation-induced aspartate-argininosuccinate shunt was identified as a link between the metabolic rewiring of the TCA cycle and the production of nitric oxide [96]. The authors developed a web-based tool, GAM (genes and metabolites), that allows integrated network analysis of transcriptional and steady-state metabolomics data and incorporates metabolic network information specific to *Arabidopsis*, yeast, mice, and humans [97]. Of note, although comprehensive, this integration approach was not a constraint-based method. In an earlier study, Shlomi et al. developed a constraint-based approach to integrate tissue-specific gene expression and protein abundance data to study the metabolic behavior of ten human tissues [98]. This approach suggested that 18% of human metabolic genes in tissues are regulated posttranscriptionally and identified tissue-specific expression of disease-associated genes such as *DLD* (dihydrolipoamide dehydrogenase) and *BCKDHA* (branched-chain keto acid dehydrogenase E1) in the brain and liver [98]. Such integrative approaches can be important for the constraint-based assessment of immune cells.

### Genome-scale metabolic modeling of patient data

This increasing understanding of the human metabolic network provides an opportunity for the clinical application of GSMMs to characterize and predict the outcome of a disease. A recent attempt to increase the patient-specific understanding of diseases is the Human Pathology Atlas developed in 2017. Based on data from ~8000 patients in 17 cancer types and datasets from local and open-source data repositories such as the Cancer Genome Atlas and the Human Protein Atlas, the authors identified genes associated with survival in various cancer types [99]. By applying GSMMs, this study identified fumarate hydratase (FH) as a conserved gene for tumor growth in all liver cancer patients, whereas succinate dehydrogenase complex subunit A (SDHA) was predicted to be important for tumor growth in 60% of patients [99]. In the case of lung cancer, SLC2A1 or GLUT1 was identified as an important marker associated with poor prognosis. This work is the first detailed attempt to study cancer-cell-type-specific and patient-specific metabolic dependencies of different cancers. In another study, a similar attempt was made to develop personalized genome-scale metabolic models for nonalcoholic fatty liver disease (NAFLD) in 45 human subjects [82]. Based on metabolic modeling, the authors identified serine deficiency as the basis of NAFLD and the serine biosynthesis pathway as a potential target for clinical interventions. Follow-up clinical studies confirmed these findings and demonstrated the health benefit of serine supplements in NAFLD patients [100]. The Human Blood Atlas has increased our understanding of gene expression in individual immune cells present in the blood [101]. Among the significant genes associated with individual immune cells, as identified by the Human Blood Atlas, are metabolic genes such as fructose transporter *SLC2A5* in B cells, aldehyde dehydrogenase 1 family member A1 (*ALDH1A1)* in monocytes, superoxide dismutase 2 (*SOD2)* in neutrophils, equilibrative nucleoside transporter 1 (ENT1 or *SLC29A1)* and catalase (*CAT*) in eosinophils, and mitochondrially encoded NADH:ubiquinone oxidoreductase core subunit 2 (*MT-ND2*) in basophils [101]. The recently released human metabolism network Human1 [88] integrates gene reaction association from the earlier reconstructions HMR 2.0 [100], Recon 3D [102] and iHSA, as well as protein complex information from Recon 3D, iHSA [103], and the CORUM [104] database. It would be interesting to apply Human1 for further analysis of human datasets to specifically study immune cell data to determine the metabolic dependencies of immune cells in various human tissues and cancer types.

## Shortcomings of current network-based modeling approaches

GSMMs and FBA make simplifying assumptions due to incomplete information. For example, computational predictions by gap-filling algorithms [105–107] are integrated into the reconstructed metabolic networks to ensure that the networks are capable of simulating desired metabolic functions. Enzyme activity measured in vitro may not reflect their catalytic activity under physiological conditions, and some enzymes may have promiscuous unannotated functions in addition to their primary functions [108]. Other simplifying assumptions, most notably the assumption of biochemical steady state, are made for computational tractability. FBA algorithms that rely on mRNA data to infer metabolic fluxes neglect the effects of posttranscriptional and posttranslational regulation and use a simplified model to determine the complex relationship between enzymes and their catalyzed reactions.

The steady-state assumption in static FBA, namely that net influx through each metabolic reaction is equal to the net outflux, hinders analysis of temporal dynamics of cellular metabolism. Some insight into the system behavior if the assumption is relaxed is offered by the dual variables of the FBA constraints, sometimes called “shadow prices” [109, 110]. Another method to overcome this caveat is to use constraint-based steady-state models with unsteady-state extensions. Bordbar et al. recently proposed unsteady state FBA (uFBA) with the goal of capturing dynamic changes in the cellular state regulated by changes in metabolism [78]. This was done by a constraint-based workflow that incorporates time-course metabolomics data in FBA to predict the metabolic flux state of a cell. This study compared FBA and uFBA methods in human red blood cells, platelets, and *Saccharomyces cerevisiae* [78]. Based on their observations, the authors propose that uFBA provides more accurate predictions than standard nondynamic FBA methods. By adding additional metabolomics data, the authors provided constraint-based modeling to study metabolic dynamics [78]. uFBA incorporates metabolite concentrations and hence paves the way for computing network flux by vertically integrating metabolomics with standard transcriptomics-based tools. However, as a shortcoming, similar to other multiomics-based approaches incorporating metabolomics, uFBA faces the challenge of measuring metabolites in rare immune cell populations specifically in disease settings. This restricts the applicability of uFBA to immune cells that can be easily obtained, cultured, and perturbed in vitro. Another approach called Single-cell Flux Estimation Analysis (scFEA) [111] relaxes the steady-state constraint by incorporating a loss term corresponding to flux imbalance into an objective function that maximizes the correlation between flux and gene expression in *supermodules* to which the metabolic network is segmented. scFEA uses a neural network to allow rich modeling of non-linear dependencies of flux on gene expression but ignores reaction stoichiometry. scFEA also segments the metabolic network and prunes non-carbon molecules, resulting in less granular mechanistic predictions [111].

GSMMs also lack widely accepted standardized protocols for workflow and the validation of model outputs. Genome-scale networks require manual and laborious curation, a process that can introduce significant variability in predictions [79, 112]. To overcome this, Richelle et al. recently developed a computational framework, CellFie, that incorporates a list of metabolic tasks alongside knowledge databases and transcriptomics data [112]. Although this work was restricted to select metabolic tasks in human transcriptomics data, this is applicable to other high-dimensional datasets from humans and other species. Further work is needed to expand the repertoire of metabolic tasks not incorporated in the study, along with the development of new methods to standardize network curation. While curation can introduce errors, the choice of a model extraction method implemented for computation can affect the outcome of GSMMs. In a study comparing the effect of six CSM methods, FASTCORE [113], GIMME [114], mCADRE [115], MBA [116], INIT [117], and iMAT [98, 118, on models of four cancer cell lines (A375, HL60, K562, and KBM7), Opdam and colleagues demonstrated that all six model extraction algorithms increased the accuracy of predictions from GSMMs; however, applied constraints, especially uptake constraints, do influence the accuracy of results [79]. Clearly, robust computational tools will be necessary to standardize the integration of gene expression and increase the prediction ability of GSMMs operated in different contexts. These will overcome the biases introduced by the workflow and constraints to allow the wider application of GSMMs.

In the domain of single-cell studies, future research will also address the lack of specificity in GSMMs. For example, different subsets of the metabolic network may be available to different cell types. The molecular composition of a cell’s environment, which is modeled in FBA and affects the outcomes of the computation, is uncertain even in synthetic and highly controlled growth environments. Better modeling of cellular environments will increase the predictive capability of FBA-based algorithms. The diverse protocols for single-cell sequencing also pose both challenges and promise. For example, we mainly tested the Compass algorithm on Smart-Seq single-cell data [3, 33]. While we showed that the predictions were reproduced in a 10x-based dataset obtained from the same model system, future studies may need to consider the protocol differences (e.g., 3’/5’ end reads vs. full transcript). In addition, the exponential growth in the size of current cell atlases will require careful consideration of computational efficiency. On the other hand, the introduction of novel assays that measure multiple data modalities opens avenues for more comprehensive modeling of cellular metabolic states than is possible with mRNA alone.

## Conclusions

The ultimate quest of all approaches for interrogating immunometabolism is finding the link between genotype and metabolic phenotype to predict patient- and disease-specific fates of immune cells and to provide novel targets to check the progression of immunological diseases. With the increased availability of large datasets, including the Human Cell Atlas [88], and the reduction in the costs of gene sequencing, the applicability of GSMMs has significantly increased in predicting the potential metabolic dependencies of cells. Immune cells respond to changes in the microenvironment such that the lineage fidelity and immune function of cells depend on available nutrients, oxygen, and tissue topology, among other parameters. This warrants a broader analysis accounting for multiple variables, such as tissue type, genetic background, age, and the sex of the individual. Increasing the comprehensiveness by incorporating specific biological constraints along with robust validation methods would therefore increase the predictive capability and applicability of GSMMs. Utilizing the outcome of such comprehensive models, one could identify strategies for modifying the function of immune cells in the context of disease. For example, by applying GSMMs to an individual’s tumor sequencing data, one could gain insights into how to develop functional effector T cells that can continue to work in the tumor microenvironment, despite the lack of certain essential nutrients or excess other metabolites that make T cells dysfunctional. Clearly, systems methods and computational models are powerful futuristic tools with their promise to predict and alter metabolic predilections of immune cells and thereby change the course of a disease.

## References

[1] Monod J. From biology to ethics. San Diego, Calif.: Salk Institute for Biological Studies; 1969.

[2] Björklund ÅK, Forkel M, Picelli S, Konya V, Theorell J, Friberg D (2016). The heterogeneity of human CD127( + ) innate lymphoid cells revealed by single-cell RNA sequencing. Nat Immunol..

[3] Gaublomme JT, Yosef N, Lee Y, Gertner RS, Yang LV, Wu C (2015). Single-cell genomics unveils critical regulators of Th17 cell pathogenicity. Cell.

[4] Gury-BenAri M, Thaiss CA, Serafini N, Winter DR, Giladi A, Lara-Astiaso D (2016). The spectrum and regulatory landscape of intestinal innate lymphoid cells are shaped by the microbiome. Cell..

[5] Jaitin DA, Kenigsberg E, Keren-Shaul H, Elefant N, Paul F, Zaretsky I (2014). Massively parallel single-cell RNA-seq for marker-free decomposition of tissues into cell types. Science.

[6] Keren-Shaul H, Spinrad A, Weiner A, Matcovitch-Natan O, Dvir-Szternfeld R, Ulland TK (2017). A unique microglia type associated with restricting development of Alzheimer’s disease. Cell.

[7] Paul F, Arkin Y, Giladi A, Jaitin DA, Kenigsberg E, Keren-Shaul H (2016). Transcriptional heterogeneity and lineage commitment in myeloid progenitors. Cell.

[8] Schlitzer A, Sivakamasundari V, Chen J, Sumatoh HR, Schreuder J, Lum J (2015). Identification of cDC1- and cDC2-committed DC progenitors reveals early lineage priming at the common DC progenitor stage in the bone marrow. Nat Immunol..

[9] Miragaia RJ, Gomes T, Chomka A, Jardine L, Riedel A, Hegazy AN (2019). Single-cell transcriptomics of regulatory T cells reveals trajectories of tissue adaptation. Immunity.

[10] Eisenstein EM, Williams CB (2009). The T(reg)/Th17 cell balance: a new paradigm for autoimmunity. Pediatr Res..

[11] Lee Y, Kuchroo V (2015). Defining the functional states of Th17 cells. F1000Research.

[12] Sungnak W, Wang C, Kuchroo VK (2019). Multilayer regulation of CD4 T cell subset differentiation in the era of single cell genomics. Adv Immunol.

[13] Villani A-C, Sarkizova S, Hacohen N (2018). Systems immunology: learning the rules of the immune system. Annu Rev Immunol..

[14] Warburg O, Gawehn K, Geissler AW (1958). [Metabolism of leukocytes]. Z Naturforsch B.

[15] Liberti MV, Locasale JW (2016). The Warburg effect: how does it benefit cancer cells?. Trends Biochem Sci..

[16] DeBerardinis RJ, Chandel NS (2016). Fundamentals of cancer metabolism. Sci Adv.

[17] Reinfeld BI, Madden MZ, Wolf MM, Chytil A, Bader JE, Patterson AR (2021). Cell-programmed nutrient partitioning in the tumour microenvironment. Nature.

[18] Cao Y, Rathmell JC, Macintyre AN (2014). Metabolic reprogramming towards aerobic glycolysis correlates with greater proliferative ability and resistance to metabolic inhibition in CD8 versus CD4 T cells. PLoS ONE.

[19] Wagner A, Wang C, Fessler J, DeTomaso D, Avila-Pacheco J, Kaminski J, et al. Metabolic modeling of single Th17 cells reveals regulators of autoimmunity. Cell. 2021;184:4168–85. 10.1016/j.cell.2021.05.045 PMC8621950 34216539

[20] Palmer CS, Anzinger JJ, Butterfield TR, McCune JM, Crowe SM. A simple flow cytometric method to measure glucose uptake and glucose transporter expression for monocyte subpopulations in whole blood. J Vis Exp. 2016;114:e54255. 10.3791/54255 PMC5091835 27584036

[21] Pacella I, Procaccini C, Focaccetti C, Miacci S, Timperi E, Faicchia D (2018). Fatty acid metabolism complements glycolysis in the selective regulatory T cell expansion during tumor growth. Proc Natl Acad Sci USA.

[22] Wang W, Green M, Choi JE, Gijón M, Kennedy PD, Johnson JK (2019). CD8^+^ T cells regulate tumour ferroptosis during cancer immunotherapy. Nature.

[23] Siska PJ, Kim B, Ji X, Hoeksema MD, Massion PP, Beckermann KE (2016). Fluorescence-based measurement of cystine uptake through xCT shows requirement for ROS detoxification in activated lymphocytes. J Immunol Methods.

[24] Beckermann KE, Hongo R, Ye X, Young K, Carbonell K, Healey DC, et al. CD28 costimulation drives tumor-infiltrating T cell glycolysis to promote inflammation. JCI Insight 2020;5:e138729. 10.1172/jci.insight.138729 PMC7455120 32814710

[25] Levine LS, Hiam-Galvez KJ, Marquez DM, Tenvooren I, Madden MZ, Contreras DC (2021). Single-cell analysis by mass cytometry reveals metabolic states of early-activated CD8^+^ T cells during the primary immune response. Immunity.

[26] Hartmann FJ, Mrdjen D, McCaffrey E, Glass DR, Greenwald NF, Bharadwaj A (2021). Single-cell metabolic profiling of human cytotoxic T cells. Nat Biotechnol..

[27] Subrahmanyam PB, Maecker HT (2017). CyTOF measurement of immunocompetence across major immune cell types. Curr Protoc Cytom..

[28] Hartmann FJ, Bendall SC (2020). Immune monitoring using mass cytometry and related high-dimensional imaging approaches. Nat Rev Rheumatol..

[29] Yucel N, Wang YX, Mai T, Porpiglia E, Lund PJ, Markov G (2019). Glucose metabolism drives histone acetylation landscape transitions that dictate muscle stem cell function. Cell Rep..

[30] Ahl PJ, Hopkins RA, Xiang WW, Au B, Kaliaperumal N, Fairhurst AM (2020). Met-Flow, a strategy for single-cell metabolic analysis highlights dynamic changes in immune subpopulations. Commun Biol.

[31] Lopes N, McIntyre C, Martin S, Raverdeau M, Sumaria N, Kohlgruber AC (2021). Distinct metabolic programs established in the thymus control effector functions of γδ T cell subsets in tumor microenvironments. Nat Immunol..

[32] Kimmey SC, Borges L, Baskar R, Bendall SC (2019). Parallel analysis of tri-molecular biosynthesis with cell identity and function in single cells. Nat Commun..

[33] Wang C, Yosef N, Gaublomme J, Wu C, Lee Y, Clish CB (2015). CD5L/AIM regulates lipid biosynthesis and restrains Th17. Cell Pathogenicity Cell.

[34] Rivadeneira DB, DePeaux K, Wang Y, Kulkarni A, Tabib T, Menk AV (2019). Oncolytic viruses engineered to enforce leptin expression reprogram tumor-infiltrating T cell metabolism and promote tumor clearance. Immunity.

[35] Pérez-Pérez A, Vilariño-García T, Fernández-Riejos P, Martín-González J, Segura-Egea JJ, Sánchez-Margalet V (2017). Role of leptin as a link between metabolism and the immune system. Cytokine Growth Factor Rev.

[36] Ringel AE, Drijvers JM, Baker GJ, Catozzi A, García-Cañaveras JC, Gassaway BM (2020). Obesity shapes metabolism in the tumor microenvironment to suppress anti-tumor immunity. Cell.

[37] Xiao Z, Dai Z, Locasale JW (2019). Metabolic landscape of the tumor microenvironment at single cell resolution. Nat Commun..

[38] Fernández-García J, Franco F, Parik S, Pane AA, Broekaert D, van Elsen J, et al. CD8^+^ T cell metabolic rewiring defined by single-cell RNA-sequencing identifies a critical role of ASNS expression dynamics in T cell differentiation. bioRxiv:2021.07.27.453976 [Preprint]. 2021. Available from: 10.1101/2021.07.27.453976.

[39] Venet F, Demaret J, Blaise BJ, Rouget C, Girardot T, Idealisoa E (2017). IL-7 restores T lymphocyte immunometabolic failure in septic shock patients through mTOR activation. J Immunol..

[40] Puleston DJ, Buck MD, Klein Geltink RI, Kyle RL, Caputa G, O'Sullivan D (2019). Polyamines and eIF5A hypusination modulate mitochondrial respiration and macrophage activation. Cell Metab.

[41] Sauer U (2006). Metabolic networks in motion: 13C-based flux analysis. Mol Syst Biol..

[42] Ron-Harel N, Santos D, Ghergurovich JM, Sage PT, Reddy A, Lovitch SB (2016). Mitochondrial biogenesis and proteome remodeling promote one-carbon metabolism for T cell activation. Cell Metab.

[43] Wu L, Hollinshead K, Hao Y, Au C, Kroehling L, Ng C (2020). Niche-selective inhibition of pathogenic Th17 cells by targeting metabolic redundancy. Cell.

[44] Sheldon RD, Ma EH, DeCamp LM, Williams KS, Jones RG. Interrogating in vivo T cell metabolism in mice using stable isotope labeling metabolomics and rapid cell sorting. Nat. Protoc. 2021. 10.1038/s41596-021-00586-2. 10.1038/s41596-021-00586-2 34349284

[45] Ma EH, Verway MJ, Johnson RM, Roy DG, Steadman M, Hayes S (2019). Metabolic profiling using stable isotope tracing reveals distinct patterns of glucose utilization by physiologically activated CD8^+^ T cells. Immunity.

[46] Geiger R, Rieckmann JC, Wolf T, Basso C, Feng Y, Fuhrer T (2016). L-arginine modulates T cell metabolism and enhances survival and anti-tumor activity. Cell.

[47] Howden A, Hukelmann JL, Brenes A, Spinelli L, Sinclair LV, Lamond AI, Cantrell DA (2019). Quantitative analysis of T cell proteomes and environmental sensors during T cell differentiation. Nat Immunol..

[48] Tan H, Yang K, Li Y, Shaw TI, Wang Y, Blanco DB (2017). Integrative proteomics and phosphoproteomics profiling reveals dynamic signaling networks and bioenergetics pathways underlying T cell activation. Immunity.

[49] Wolf T, Jin W, Zoppi G, Vogel IA, Akhmedov M, Bleck C (2020). Dynamics in protein translation sustaining T cell preparedness. Nat Immunol..

[50] Ghergurovich JM, García-Cañaveras JC, Wang J, Schmidt E, Zhang Z, TeSlaa T (2020). A small molecule G6PD inhibitor reveals immune dependence on pentose phosphate pathway. Nat Chem Biol..

[51] Huang H, Zhou P, Wei J, Long L, Shi H, Dhungana Y (2021). In vivo CRISPR screening reveals nutrient signaling processes underpinning CD8^+^ T cell fate decisions. Cell.

[52] Fu G, Guy CS, Chapman NM, Palacios G, Wei J, Zhou P (2021). Metabolic control of TFH cells and humoral immunity by phosphatidylethanolamine. Nature.

[53] Wei J, Long L, Zheng W, Dhungana Y, Lim SA, Guy C (2019). Targeting REGNASE-1 programs long-lived effector T cells for cancer therapy. Nature.

[54] Katzenelenbogen Y, Sheban F, Yalin A, Yofe I, Svetlichnyy D, Jaitin DA (2020). Coupled scRNA-seq and intracellular protein activity reveal an immunosuppressive role of TREM2 in cancer. Cell.

[55] Hu KH, Eichorst JP, McGinnis CS, Patterson DM, Chow ED, Kersten K (2020). ZipSeq: barcoding for real-time mapping of single cell transcriptomes. Nat Methods.

[56] O’Neill LAJ, Kishton RJ, Rathmell J (2016). A guide to immunometabolism for immunologists. Nat Rev Immunol..

[57] Voss K, Hong HS, Bader JE, Sugiura A, Lyssiotis CA, Rathmell JC (2021). A guide to interrogating immunometabolism. Nat Rev Immunol..

[58] Singer BD, Chandel NS (2019). Immunometabolism of pro-repair cells. J Clin Investig..

[59] Roy DG, Kaymak I, Williams KS, Ma EH, Jones RG (2021). Immunometabolism in the tumor microenvironment. Annu Rev Cancer Biol.

[60] Pålsson-McDermott EM, O’Neill LAJ (2020). Targeting immunometabolism as an anti-inflammatory strategy. Cell Res.

[61] Kanehisa M, Goto S, Sato Y, Furumichi M, Tanabe M (2012). KEGG for integration and interpretation of large-scale molecular data sets. Nucleic Acids Res.

[62] Schilling CH, Letscher D, Palsson BO (2000). Theory for the systemic definition of metabolic pathways and their use in interpreting metabolic function from a pathway-oriented perspective. J Theor Biol..

[63] Stelling J, Klamt S, Bettenbrock K, Schuster S, Gilles ED (2002). Metabolic network structure determines key aspects of functionality and regulation. Nature.

[64] Nielsen J (2017). Systems biology of metabolism. Annu Rev Biochem.

[65] Jahan N, Maeda K, Matsuoka Y, Sugimoto Y, Kurata H (2016). Development of an accurate kinetic model for the central carbon metabolism of *Escherichia coli*. Microb Cell Fact.

[66] Teusink B, Passarge J, Reijenga CA, Esgalhado E, van der Weijden CC, Schepper M (2000). Can yeast glycolysis be understood in terms of in vitro kinetics of the constituent enzymes? Testing biochemistry. Eur J Biochem.

[67] Smallbone K, Messiha HL, Carroll KM, Winder CL, Malys N, Dunn WB (2013). A model of yeast glycolysis based on a consistent kinetic characterisation of all its enzymes. FEBS Lett.

[68] Bordbar A, McCloskey D, Zielinski DC, Sonnenschein N, Jamshidi N, Palsson BO (2015). Personalized whole-cell kinetic models of metabolism for discovery in genomics and pharmacodynamics. Cell Syst.

[69] Yurkovich JT, Yang L, Palsson BO. Systems-level physiology of the human red blood cell is computed from metabolic and macromolecular mechanisms. bioRxiv:797258 [Preprint] 2019. Available from: 10.1101/797258.

[70] Nilsson A, Nielsen J (2017). Genome scale metabolic modeling of cancer. Metab Eng.

[71] O’Brien EJ, Monk JM, Palsson BO (2015). Using genome-scale models to predict biological capabilities. Cell.

[72] Cook DJ, Nielsen J (2017). Genome-scale metabolic models applied to human health and disease. Wiley Interdiscip Rev: Syst Biol Med.

[73] Palsson B (2004). Two-dimensional annotation of genomes. Nat Biotechnol..

[74] Reed JL, Famili I, Thiele I, Palsson BO (2006). Towards multidimensional genome annotation. Nat Rev Genet..

[75] Jamshidi N, Palsson BØ (2010). Mass action stoichiometric simulation models: incorporating kinetics and regulation into stoichiometric models. Biophys J..

[76] Burgard AP, Pharkya P, Maranas CD (2003). Optknock: a bilevel programming framework for identifying gene knockout strategies for microbial strain optimization. Biotechnol Bioeng..

[77] Feist AM, Palsson BO (2010). The biomass objective function. Curr Opin Microbiol..

[78] Bordbar A, Yurkovich JT, Paglia G, Rolfsson O, Sigurjónsson ÓE, Palsson BO (2017). Elucidating dynamic metabolic physiology through network integration of quantitative time-course metabolomics. Sci Rep..

[79] Opdam S, Richelle A, Kellman B, Li S, Zielinski DC, Lewis NE (2017). A systematic evaluation of methods for tailoring genome-scale metabolic models. Cell Syst.

[80] Hyötyläinen T, Jerby L, Petäjä EM, Mattila I, Jäntti S, Auvinen P (2016). Genome-scale study reveals reduced metabolic adaptability in patients with non-alcoholic fatty liver disease. Nat Commun..

[81] Wu H-Q, Cheng ML, Lai JM, Wu HH, Chen MC, Liu WH (2017). Flux balance analysis predicts Warburg-like effects of mouse hepatocyte deficient in miR-122a. PLoS Comput Biol..

[82] Mardinoglu A, Agren R, Kampf C, Asplund A, Nookaew I, Jacobson P (2013). Integration of clinical data with a genome-scale metabolic model of the human adipocyte. Mol Syst Biol..

[83] Ramirez AK, Lynes MD, Shamsi F, Xue R, Tseng YH, Kahn CR (2017). Integrating extracellular flux measurements and genome-scale modeling reveals differences between brown and white adipocytes. Cell Rep..

[84] Echeverri-Peña OY, Salazar-Barreto DA, Rodríguez-Lopez A, González J, Alméciga-Díaz CJ, Verano-Guevara CH (2021). Use of a neuron-glia genome-scale metabolic reconstruction to model the metabolic consequences of the Arylsulphatase a deficiency through a systems biology approach. Heliyon.

[85] Martín-Jiménez CA, Salazar-Barreto D, Barreto GE, González J (2017). Genome-scale reconstruction of the human astrocyte metabolic network. Front Aging Neurosci..

[86] Bordbar A, Mo ML, Nakayasu ES, Schrimpe-Rutledge AC, Kim YM, Metz TO (2012). Model-driven multi-omic data analysis elucidates metabolic immunomodulators of macrophage activation. Mol Syst Biol..

[87] Hörhold F, Eisel D, Oswald M, Kolte A, Röll D, Osen W (2020). Reprogramming of macrophages employing gene regulatory and metabolic network models. PLoS Comput Biol..

[88] Robinson JL, Kocabaş P, Wang H, Cholley PE, Cook D, Nilsson A, et al. An atlas of human metabolism. Sci Signal. 2020;13:eaaz1482. 10.1126/scisignal.aaz1482 PMC7331181 32209698

[89] Baran Y, Bercovich A, Sebe-Pedros A, Lubling Y, Giladi A, Chomsky E (2019). MetaCell: analysis of single-cell RNA-seq data using K-nn graph partitions. Genome Biol.

[90] Giladi A, Cohen M, Medaglia C, Baran Y, Li B, Zada M (2020). Dissecting cellular crosstalk by sequencing physically interacting cells. Nat Biotechnol..

[91] Angelin A, Gil-de-Gómez L, Dahiya S, Jiao J, Guo L, Levine MH (2017). Foxp3 reprograms T cell metabolism to function in low-glucose, high-lactate environments. Cell Metab.

[92] Pucino V, Certo M, Bulusu V, Cucchi D, Goldmann K, Pontarini E (2019). Lactate buildup at the site of chronic inflammation promotes disease by inducing CD4^+^ T cell metabolic rewiring. Cell Metab.

[93] Bordbar A, Monk JM, King ZA, Palsson BO (2014). Constraint-based models predict metabolic and associated cellular functions. Nat Rev Genet..

[94] Zhang Y, Kim MS, Nguyen E, Taylor DM. Modeling metabolic variation with single-cell expression data. bioRxiv:2020.01.28.923680 [Preprint] 2020. Available from: 10.1101/2020.01.28.923680.

[95] Yilmaz LS, Li X, Nanda S, Fox B, Schroeder F, Walhout AJ (2020). Modeling tissue-relevant *Caenorhabditis elegans* metabolism at network, pathway, reaction, and metabolite levels. Mol Syst Biol..

[96] Jha AK, Huang SC, Sergushichev A, Lampropoulou V, Ivanova Y, Loginicheva E (2015). Network integration of parallel metabolic and transcriptional data reveals metabolic modules that regulate macrophage polarization. Immunity.

[97] Sergushichev AA, Loboda AA, Jha AK, Vincent EE, Driggers EM, Jones RG (2016). GAM: a web-service for integrated transcriptional and metabolic network analysis. Nucleic Acids Res.

[98] Shlomi T, Cabili MN, Herrgård MJ, Palsson BØ, Ruppin E (2008). Network-based prediction of human tissue-specific metabolism. Nat Biotechnol..

[99] Uhlen M, Zhang C, Lee S, Sjöstedt E, Fagerberg L, Bidkhori G, et al. A pathology atlas of the human cancer transcriptome. Science. 2017;357:eaan2507. 10.1126/science.aan2507 28818916

[100] Mardinoglu A, Agren R, Kampf C, Asplund A, Uhlen M, Nielsen J (2014). Genome-scale metabolic modelling of hepatocytes reveals serine deficiency in patients with non-alcoholic fatty liver disease. Nat Commun..

[101] Uhlen M, Karlsson MJ, Zhong W, Tebani A, Pou C, Mikes J, et al. A genome-wide transcriptomic analysis of protein-coding genes in human blood cells. Science. 2019;366:eaax9198. 10.1126/science.aax9198 31857451

[102] Brunk E, Sahoo S, Zielinski DC, Altunkaya A, Dräger A, Mih N (2018). Recon3D enables a three-dimensional view of gene variation in human metabolism. Nat Biotechnol..

[103] Blais EM, Rawls KD, Dougherty BV, Li ZI, Kolling GL, Ye P (2017). Reconciled rat and human metabolic networks for comparative toxicogenomics and biomarker predictions. Nat Commun..

[104] Ruepp A, Brauner B, Dunger-Kaltenbach I, Frishman G, Montrone C, Stransky M (2008). CORUM: the comprehensive resource of mammalian protein complexes. Nucleic Acids Res.

[105] Hatzimanikatis V, Li C, Ionita JA, Henry CS, Jankowski MD, Broadbelt LJ (2005). Exploring the diversity of complex metabolic networks. Bioinformatics.

[106] Herrgård MJ, Fong SS, Palsson BØ (2006). Identification of genome-scale metabolic network models using experimentally measured flux profiles. PLoS Comput Biol..

[107] Satish Kumar V, Dasika MS, Maranas CD (2007). Optimization based automated curation of metabolic reconstructions. BMC Bioinforma.

[108] Guzmán GI, Sandberg TE, LaCroix RA, Nyerges Á, Papp H, de Raad M (2019). Enzyme promiscuity shapes adaptation to novel growth substrates. Mol Syst Biol..

[109] Reznik E, Mehta P, Segrè D (2013). Flux imbalance analysis and the sensitivity of cellular growth to changes in metabolite pools. PLoS Comput Biol..

[110] Wagner A, Zarecki R, Reshef L, Gochev C, Sorek R, Gophna U (2013). Computational evaluation of cellular metabolic costs successfully predicts genes whose expression is deleterious. Proc Natl Acad Sci USA..

[111] Alghamdi N, Chang W, Dang P, Lu X, Wan C, Gampala S (2021). A graph neural network model to estimate cell-wise metabolic flux using single-cell RNA-seq data. Genome Res..

[112] Richelle A, Kellman BP, Wenzel AT, Chiang A, Reagan T, Gutierrez JM (2021). Model-based assessment of mammalian cell metabolic functionalities using omics data. Cell Rep Methods.

[113] Vlassis N, Pacheco MP, Sauter T (2014). Fast reconstruction of compact context-specific metabolic network models. PLoS Comput Biol..

[114] Becker SA, Palsson BO (2008). Context-specific metabolic networks are consistent with experiments. PLoS Comput Biol..

[115] Wang Y, Eddy JA, Price ND (2012). Reconstruction of genome-scale metabolic models for 126 human tissues using mCADRE. BMC Syst Biol..

[116] Jerby L, Shlomi T, Ruppin E (2010). Computational reconstruction of tissue-specific metabolic models: application to human liver metabolism. Mol Syst Biol..

[117] Agren R, Bordel S, Mardinoglu A, Pornputtapong N, Nookaew I, Nielsen J (2012). Reconstruction of genome-scale active metabolic networks for 69 human cell types and 16 cancer types using INIT. PLoS Comput Biol..

[118] Zur H, Ruppin E, Shlomi T (2010). iMAT: an integrative metabolic analysis tool. Bioinformatics.

[119] Wang K, Li M, Hakonarson H (2010). Analysing biological pathways in genome-wide association studies. Nat Rev Genet..

[120] Maleki F, Ovens K, Hogan DJ, Kusalik AJ (2020). Gene set analysis: challenges, opportunities, and future research. Front Genet..

[121] Ashburner M, Ball CA, Blake JA, Botstein D, Butler H, Cherry JM (2000). Gene Ontology: tool for the unification of biology. Nat Genet..

[122] Gene Ontology Consortium. (2015). Gene Ontology Consortium: going forward. Nucleic Acids Res.

[123] Bauer E, Thiele I. From network analysis to functional metabolic modeling of the human gut microbiota. mSystems. 2018;3:e00209-17. 10.1128/mSystems.00209-17 PMC5872302 29600286

[124] Fang X, Lloyd CJ, Palsson BO (2020). Reconstructing organisms in silico: genome-scale models and their emerging applications. Nat Rev Microbiol..

[125] Orth JD, Thiele I, Palsson BØ (2010). What is flux balance analysis?. Nat Biotechnol..

[126] Lewis NE, Hixson KK, Conrad TM, Lerman JA, Charusanti P, Polpitiya AD (2010). Omic data from evolved *E. coli* are consistent with computed optimal growth from genome-scale models. Mol Syst Biol..

[127] Segre D, Vitkup D, Church GM (2002). Analysis of optimality in natural and perturbed metabolic networks. Proc Natl Acad Sci USA.

[128] Yizhak K, Gabay O, Cohen H, Ruppin E (2013). Model-based identification of drug targets that revert disrupted metabolism and its application to ageing. Nat Commun..

[129] Wagner A, Regev A, Yosef N (2016). Revealing the vectors of cellular identity with single-cell genomics. Nat Biotechnol..

[130] van Dijk D, Sharma R, Nainys J, Yim K, Kathail P, Carr AJ (2018). Recovering gene interactions from single-cell data using data diffusion. Cell.

[131] Haghverdi L, Lun ATL, Morgan MD, Marioni JC (2018). Batch effects in single-cell RNA-sequencing data are corrected by matching mutual nearest neighbors. Nat Biotechnol..

[132] Huang M, Wang J, Torre E, Dueck H, Shaffer S, Bonasio R (2018). SAVER: gene expression recovery for single-cell RNA sequencing. Nat Methods.

[133] Lun ATL, Bach K, Marioni JC (2016). Pooling across cells to normalize single-cell RNA sequencing data with many zero counts. Genome Biol.

[134] DeTomaso D, Jones MG, Subramaniam M, Ashuach T, Ye CJ, Yosef N (2019). Functional interpretation of single cell similarity maps. Nat Commun..

